# Valence band behaviour of zirconium oxide, Photoelectron and Auger spectroscopy study

**DOI:** 10.1038/s41598-018-34570-w

**Published:** 2018-11-02

**Authors:** Zakaria Azdad, Laurent Marot, Lucas Moser, Roland Steiner, Ernst Meyer

**Affiliations:** Department of Physics, University of Basel, Klingbergstrasse 82, 4056 Basel, Switzerland

## Abstract

In this study X-ray Photoelectron Spectroscopy and Ultraviolet Photoelectron Spectroscopy were combined to investigate the effect of oxygen incorporation on the valence band behaviour of ZrO_*x*_. The Auger transitions involving valence bands are found to mimic the self-folded density of state measured using Ultraviolet Photoelectron Spectroscopy. The valence band once constructed in a sub-oxide form, stays at a fixed energy position despite the change in the stoichiometry. This behaviour is found to be useful in setting a reference for X-ray Photoelectron Spectroscopy charge correction. The results of the charged corrected spectra were compared to other methods and found to be in great agreement. Finally, a correlation between the core-level binding energy and the structural property of ZrO_*x*_ is given.

## Introduction

Zirconium dioxide (ZrO_2_) has been studied extensively due to its physical and chemical properties, whether in high temperature fuel cells^1–3^, gas sensors^4–6^, protective coating for metal anti-corrosion^7–9^, or insulator on metal-oxide semiconductor devices^10–12^. ZrO_2_ still attracts attention of other fields, thus the understanding of its electronic behaviour is crucial for the development of various technology sectors. Using photo-electron spectroscopy techniques to study the properties of such an insulator is a challenging task. Indeed, the build-up of static charges at the surface for thicknesses above 5 nm can randomly shift the measured spectra^13,14^ depending on the thickness, micro-structure of the film and exposure time to X-ray photoelectron spectroscopy (XPS) excitation. As an example, we mention that the reported band gap value of ZrO_2_ varied from 3.8 to 5.8 eV for the monoclinic phase depending on the measurement techniques^15–18^. This large range between reported values has been puzzling both experimentalists and theoreticians and no clear explanation has been given up-to date^19^.

Although previous studies already addressed surface oxidation of metallic Zr under various oxygen atmosphere using photo-electron spectroscopy techniques^20–23^, they did not provide a full interpretation of the valence behaviour of *ZrO*_*x*_ films. In the current investigation, the oxidation state of the films as well as their thicknesses have been controlled directly by reactive magnetron sputtering. This method allowed us to avoid the charging effect and to eliminate the contribution of the substrate to the measured signal. The study of Auger, core-level and valence spectra using XPS and Ultraviolet Photoelectron Spectroscopy (UPS) of the *ZrO*_*x*_ films showed that the variation of the oxidation state do not influence the position of the O2p bands but rather the band edge of the upper valence band. Auger transitions involving valence bands could therefore be used as a reference for charge correction of the XPS spectra and give a better understanding of the evolution of the valence bands upon oxidation. Based on Auger transition corrections, an attempt to correlate the obtained results with the structural properties of ZrO_*x*_ will be presented.

## Results and Discussion

**Figure 1:**
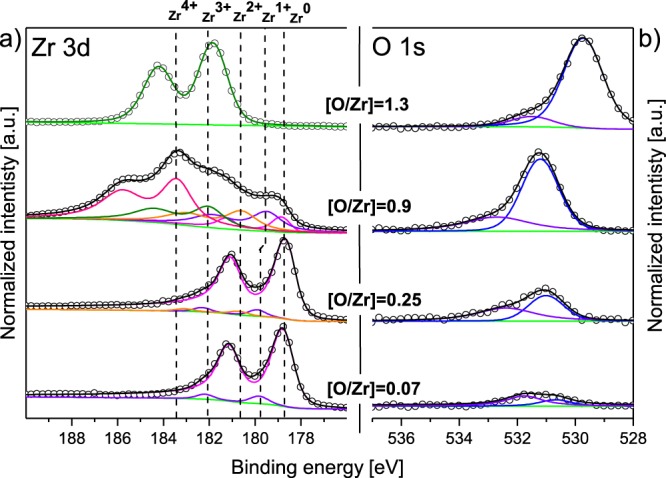
(**a**) Core level Zr3d spectra (XPS) for bulky (≥40 nm) metallic $([\frac{O}{Zr}]=0.07)$ and partially oxidized films $([\frac{O}{Zr}]=0.25\,{\rm{and}}\,[\frac{O}{Zr}]=0.9)$ as well as for thin oxide films $5\,{\rm{m}}{\rm{n}},([\frac{O}{Zr}]=1.3)$ with different oxidation states: Zr^0^ for metallic, Zr^1+^, Zr^2+^, Zr^3+^ for sub-oxides and Zr^4+^ for stoichiometric oxide. (**b**) Core level O1s spectra (XPS) for the corresponding Zr3d spectra. The open circles stand for the measured spectrum and the black lines correspond to the sum curve of all components represented in the coloured lines. The vertical lines are given as a guide to the eye.

In order to investigate the valence band behaviour and the Auger transitions of ZrO_*x*_ as a function of oxygen incorporation, several thin film samples were prepared with magnetron sputtering under different argon/oxygen atmosphere that led to various $\frac{O}{Zr}$ ratios (calculated from XPS measurements). In a first stage, bulky films (≥40 nm) were prepared and analysed by *in situ* XPS and UPS measurements (without breaking the vacuum). A Zr film was first deposited in a pure argon atmosphere and the core-level spectra (Zr3d and O1s) of this metallic film $(\frac{O}{Zr}=0.07)$ are presented in Fig. 1 along with other $\frac{O}{Zr}$ ratios. As can be seen, the measured core-level spectrum of Zr3d was deconvoluted into two doublets as described by Morant *et al*.^24^ which correspond to Zr in metallic state (Zr^0^) at 178.7 eV and to a sub-oxide (Zr^1+^) located at 179.8 eV binding energy (BE). For the metallic component, due to the continuous distribution of states across the Fermi level (E_*F*_), the asymmetric tail at high energy should be taken into account when fitting the XPS spectrum. In our case, an asymmetry factor was fixed at 0.1, slightly lower than the value reported by Ma *et al*.^25^ and Lyapin *et al*.^26^ (0.13 and 0.18, respectively). Figure 1b displays the as-measured intensity of O1s core-level. The weak photo-emission intensity confirms the metallic feature of the deposited film which is only composed of 7 at.% oxygen. Nevertheless for an accurate fitting of the measured spectrum, both absorbed oxygen and oxide component were considered, with BE determined at 531.7 and 530.4 eV respectively (see Fig. 1b).

**Figure 2:**
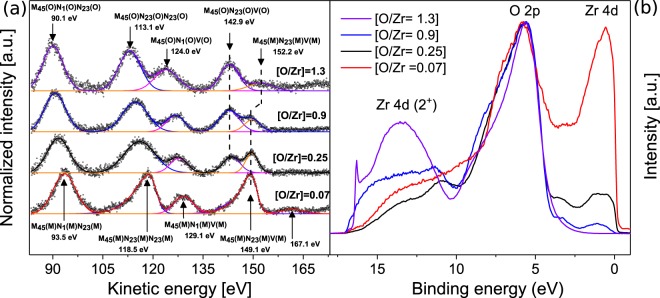
(**a**) As measured Auger spectra for the indicated $\frac{O}{Zr}$ ratios with arbitrary vertical shift where the inserted arrows indicate the KE of the Auger transitions. The colored lines stand for the different components. The open circles are the spectrum data points after background subtraction. The solid lines behind the open circles represent the sum of the different components. (**b**) Normalized UPS spectra for the corresponding Auger spectra using helium I (HeI).

The Auger spectrum of the metallic film $([\frac{O}{Zr}]=0.07)$ is presented in red in Fig. 2a along with other $\frac{O}{Zr}$ ratios. As can be seen, the spectrum is composed of four main transitions: M_45_(M)N_1_(M)N_23_(M), M_45_(M)N_23_(M)N_23_(M), M_45_(M)N_1_(M)V(M) and M_45_(M)N_23_(M)V(M) where the M in brackets stands for metallic, and located in the energy range of the reported values from literature^27–29^. In addition to these main transitions, a weak peak is observed at 161.7 eV and its origin is not determined (same peak has been observed by Tunable *et al*.^30^). The UPS spectrum of the same metallic film (red curve, Fig. 1b) exhibits an O2p and Zr4d band of equivalent intensity due to the high photo-ionization cross section of oxygen^31^.

In a second step, a partially oxidized film was deposited by adding oxygen to the argon atmosphere with a composition $\frac{O}{Zr}$ = 0.25. The measured core-level spectrum of Zr3d (see Fig. 1a) shows a dominant peak of Zr^0^ state as well as two smaller peaks attributed to sub-oxides (Zr^1+^ and Zr^2+^), indicating that the film still possesses metallic features but contains more oxidized Zr compared to the previous film. The measured core level of O1s can, as above, be deconvoluted into two peaks located at 531 and 532.4 eV BE attributed to Zr-O bonding and adsorbed oxygen respectively (see Fig. 1b). The recorded Auger spectrum of the partially oxide film with $\frac{O}{Zr}$ = 0.25 displays a splitting of M_45_(M)N_23_(M)V(M) into M_45_(M)N_23_(M)V(M) and M_45_(O)N_23_(O)V(O) (where O stands for oxide). These transitions are located at 149.4 and 143.5 eV KE and are related to the electronic transitions from Zr4d and O2p orbitals observed in the valence spectrum (see Fig. 2b). On one hand, M_45_(M)N_23_(M)V(M) transition has a sharp peak assigned to transitions involving Zr4d orbitals. On the other hand the broad peak attributed to M_45_(O)N_23_(O)V(O) is a consequence of the electronic transitions from the broad O2p non-bounding and O-cation hybridized bonding orbitals. The difference in energy between these transitions is 5.9 eV, in agreement with the value known for metallic Zr surface exposed to low oxygen concentration (5.7 eV^32^). For the other transitions, a shift of 2 eV towards lower KE is observed. Regarding the UPS spectrum shown in Fig. 2b, one can notice the presence of a shoulder next to the O2p band located around 3.2 eV. This shoulder is attributed to oxygen defect states^33^ and is also observed for $\frac{O}{Zr}$ = 0.9.

Increasing the $\frac{O}{Zr}$ ratio influences the Auger transitions due to the change in the chemical environment that Zr atoms experience. The Auger spectra for $\frac{O}{Zr}$ = 0.9 shows a shift between 2.5 and 3.8 eV towards lower KE for transitions involving core levels (CCC for core-core-core) with respect to pure metallic film, while the position of the CCV (Core-core-valence) transition remains fairly unchanged (see Fig. 2a). Furthermore, from the valence band spectrum we can notice an increase in the O2p density of state and a decrease in the Zr4d density of state (see Fig. 2b). This population inversion is also observed in the measured intensity of the CCV transitions where the M_45_(O)N_23_(O)V(O) intensity becomes higher than the M_45_(M)N_23_(M)V(M) intensity, highlighting the formation of ionic bonding with oxygen by increasing the oxygen content. This observation was confirmed with XPS measurements as the Zr3d core-level spectrum (see Fig. 1a) exhibits five components among which four are attributed to oxidized species (three sub-oxides and one stoichiometric oxide located at 179.5, 180.9, 182.2 and 183.5 eV BE respectively). Yet, as a metallic component of Zr remained distinguishable both in UPS (Zr4d) and in XPS (Zr^0^), no shifts due to charging are expected.

Further increase in the $\frac{O}{Zr}$ ratio led to strong variations of the measured Auger and core-level spectra which make their interpretation controversial without accounting for sample charging. In order to eliminate the charging effect, a very thin film of about 5 nm and $\frac{O}{Zr}$ = 1.3 was employed. UPS and XPS measurement were systematically performed and the results were compared to metallic and partially oxidized zirconium films $(\frac{O}{Zr}=0.07,0.25\,{\rm{and}}\,0.09)$. As can be seen from the XPS measurement (see Fig. 1a), it was not possible to deposit a stoichiometric film at room temperature with the present setup (Zr^2+^ and Zr^3+^ sub-oxides). Regarding the O1s core level spectrum, the peaks are shifted compared to previous measurements and it could originate from the complete oxidation of the film. From UPS measurement (see Fig. 2b), the increase in the oxygen concentration shows no variation of the O2p bands position located at 5.6 eV below the Fermi level. Despite the non-stoichiometry of the film in this work, the same O2p band position was found in the UPS and XPS analysis of ZrO_2_ performed by Sayan *et al*. in^34^, confirming the validity of the present study. Going back to the analysis of Fig. 2b, the intensity of the metallic Zr4d band at the Fermi edge was found to diminish with increasing $\frac{O}{Zr}$ ratio. Simultaneously, a broad peak centered at 13.5 eV below the Fermi level appeared and was attributed to Zr4d (2+) transitions^17^. Furthermore, the shoulder attributed to oxygen defect states at 3.2 eV completely vanished as enough oxygen was available during the growth process. Also the width of the O2p O-cation hybridized bonding orbitals becomes narrower as the incorporated oxygen increases, while O2p non-bounding orbitals show a decrease in their intensity indicating the formation of strongly bounded Zr-O.

Comparing the Auger and the UPS spectra from Fig. 2, one can note that the M_45_(M)N_23_(M)V(M) and M_45_(O)N_23_(O)V(O) are mimicking the Zr4d and O2p valence density of state (V-DOS) respectively. The first transition shows a decrease of its intensity and vanishes as the amount of oxygen increases. The second shows a relatively small increase of its intensity without shifting the position. An other interesting feature is the non equal shift in the CCC transitions for $\frac{O}{Zr}$ = 1.3 film. The M_45_(O)N_1_(O)N_23_(O) and M_45_(O)N_23_(O)N_23_(O) are shifted of about 3.6 and 5.6 eV respectively. This difference suggests a variation in the interactions between Zr-O and O-O with increasing oxygen content^30^. Since the O2s bands are closer in energy to Zr4p than with Zr4s, the overlap of O2s wave function with Zr4p is much stronger than Zr4s and can explain the non-equal shift in Auger transitions. The small peak existing at 152.2 eV KE was referred to an Auger peak of sulfur (S) contamination by Vaughan *et al*. in^35^. However, in our case XPS dedicated scans revealed no trace of neither S2p nor S2s core-level. We believe that this peak could originate from Auger recombination process, that can results in emission of electrons from the conduction band.

The combined analysis of UPS and Auger spectra has proven that increasing the $\frac{O}{Zr}$ ratio has no effect on the position of the O2p valence band and thus on the Auger transitions involving valence bands M_45_(M)N_23_(M)V(M), which could therefore be taken as a reference for charge correction of the spectra.

To visualize the effect of the sample charging, different thick films (≈40 nm) were deposited with radio-frequency (RF) magnetron sputtering under different conditions, insuring the same stoichiometry but different surface roughness measured using non contact atomic force microscopy (see Figure S1 Supplementary Information). X-ray diffractometer (XRD) measurements have confirmed that all the films have monoclinic phase with crystallite size around 5 nm (calculated by the Scherrer’s formula^36^) oriented along [−111] direction (see Figure S2 Supplementary Information).

The behaviour of the MNV transitions discussed before was implemented to correct the shift of the measured core-level spectra due to charging in bulk films. From the as measured spectra, the effect of the surface charging shows a random shift of the Zr3d core-level (see Figure S3a Supplementary Information). Correcting the BE of Zr3d using MNV transition as a reference gave the same value of the BE that is commonly reported in the literature and attributed to non-stoichiometric state of zirconium. The ratio $\frac{O}{Zr}$ = 1.3 shows indeed that the deposited films are in a sub-oxide form (see Figure S3b of supplementary information). To verify the implemented method, we exposed one of the deposited films to air and we measured the core-levels of C, O and Zr. The C1s core-level was measured at 287.4 eV, higher than the usually adsorbed carbon located at 285 eV. The Zr3d peak was measured at 184.2 eV, which exceeds the binding energy of zirconium, while O1s was measured at 532 eV. By using C1s BE 285 eV for charge correction^37,38^, the Zr3d is found at 181.6 and O1s at 529.4 eV (see Table 1). The error of ±0.2 eV of the corrected BE is due to the C1s peak reference position that can be found in the literature. Note also that the BE of the core-level O1s before and after exposing the sample to air is not the same. This difference is due to formation of carbonyl/C=O bounds that appear clearly from the core-level spectra of both C1s and O1s (see Figure S4 of Supplementary Information). To further check the validity of this method, we deposited a thick film of 54 nm of ZrO_1.3_ and a systematic measurement of both core-level and Auger spectra was performed. The binding energy of both Zr3d and O1s were located at 183.4 and 531.4 eV, respectively. The MNV transitions from the Auger spectra is found at 141.8 eV KE, meaning that the spectrum is shifted by 1.7 eV. Applying this shift to the core-levels, Zr3d and O1s are found at 181.7 and 529.7 eV respectively. To verify this hypothesis a gold (Au) thin film of about 1 nm was deposited on the same sample without breaking the vacuum. The peak position of Au4f was found at 86.3 eV (see Figure S5a of Supplementary Information). The BE of Zr3d and O1s were measured at 184.1 and at 532.1 eV respectively (see Figure S5b,c of Supplementary Information). If one applies the shift correction using Au4f peak at 84 eV, then the Zr3d and O1s peak are at 181.8 and 529.8 eV (see Table 1). The striking agreement between the two method is obvious.

**Table 1:** BE in eV before and after charge correction for carbon exposed film and Au film deposited on the top.

		Zr3d	O1s	C1s/Au4f
Air exposure	As deposited	184.2	532	—
	After exposure to air	184.2	532.2	287.4
	After charge correction using C1s peak	181.6	529.4	285
	After charge correction using MNV transitions	181.8	529.8	—
Au thin film	As deposited	183.4	531.4	—
	Au film on the top	184.1	532.1	86.3
	After charge correction using Au4f peak	181.8	529.8	84
	After charge correction using MNV transitions	181.7	529.7	—

**Figure 3:**
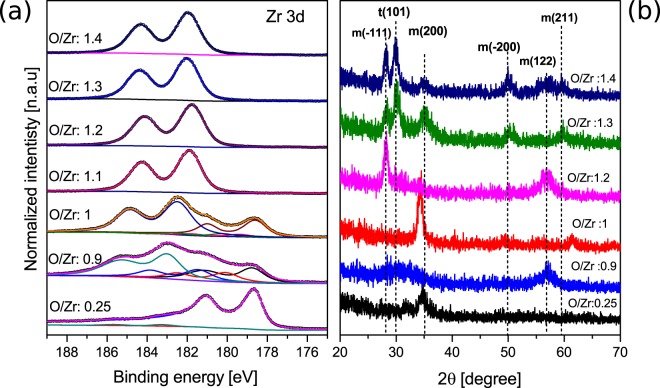
(**a**) Core level Zr3d spectra (XPS) for bulky (≥80 nm) films corrected using MNV reference. The colored lines are the different components. The open circles are the spectrum data points after background subtraction. The solid line behind the open circles is the fit sum of the components. (**b**) XRD spectra of several deposited film with different stoichiometry (t for tetragonal phase, m for monoclinic).

Now that we can correct the charging of our sample properly we can attribute the shift in BE to changes of the film morphology. *Ex-situ* XRD measurements were performed on thin films with different $\frac{O}{Zr}$ ratios, thicknesses around 80 nm and shown in Fig. 3b, where the thickness is measured by scanning electron microscopy (SEM) cross-section (see Figure S6 of Supplementary Information). A general trend of changing the structure from nearly amorphous to highly crystalline structure is accompanied by a change in the crystalline orientation and appearance of new phases upon increasing oxygen concentration in the film. Furthermore, the binding energy of stoichiometric component of ZrO_*x*_ moves toward lower BE as the transition from poorly amorphous film to crystalline film occurs. This BE shift of the Zr3d_5/2_ can only be due to the change in the structure since the increase of the oxidation state of Zr does not influence the position of the core level of the oxide component^24^. The increase in the $\frac{O}{Zr}$ ratio from 1 to 1.2 induces a change in the crystalline orientation and disappearance of the Zr3d metallic component. Due to the shift observed in the measured spectra, we applied the same correction method used before. Both Zr3d core level BE (181.9 eV) and crystalline structure are similar to the sub-oxides obtained before, confirming that the increase in the $\frac{O}{Zr}$ ratio with conservation of the crystalline structure has no effect on the BE. Another interesting fact is that for $\frac{O}{Zr}$ = 1.3 with multi-crystalline phase, the Zr3d binding energy after correction is found at 182.1 eV. This slight difference in the binding energy can be explained by the change in the work function due to inhomogeneous surface energies caused by the presence of different crystalline phase. The further increase of the $\frac{O}{Zr}$ ratio to 1.4 does not affect the binding energy as the crystalline structure is conserved.

## Conclusions

The correlation between the Auger transitions and the valence band with increasing oxygen content in zirconium has been addressed. The localized O2p band and the MNV transition have served as reference for charge correction of the XPS spectra due to sample charging. This approach was compared to the commonly used methods either by exposing the sample to air or by depositing an Au thin film on top of the samples. While these methods do not allow to study the evolution of the electronic structure without contamination of the sample, the new implemented approach provides a good understanding of the electronic behaviour of zirconium under different oxides form and micro-structure. Especially, it was proven that the binding energy of ZrO_*x*_ moves toward lower BE due to a transition from poorly amorphous film to crystalline film with increasing oxygen content. The behaviour of the valence band with increasing oxygen content for zirconium dioxide could also be effective to study similar type of oxide families such as TiO_2_ or HfO_2_.

## Methods

Different thin films were prepared using pulsed direct current (DC) or radio frequency magnetron sputtering under different argon/oxygen atmosphere. The description of the setup can be found elsewhere^39^. The samples were transferred from the high vacuum plasma chamber to the ultra-high vacuum chamber housing a photo-electron spectrometer without breaking the vacuum. The electron spectrometer is equipped with a hemispherical analyzer (Leybold EA10/100 MCD) and a non-monochromatized magnesium K_*α*_ X-ray source (*hν* = 1253.6 eV) was used for core level spectroscopy. The binding energy scale was calibrated using the Au4f_7/2_ line of a cleaned Au sample at 84.0 eV. The acquisition mode was set to constant analyzer energy with 29 eV pass energy (0.05 eV step size) and normal electron escape angle. The typical resolution is 0.8 eV. The deconvolution of the spectra was performed using Unifit software package^40^. For UPS measurements, a helium discharge lamp emitting in ultraviolet range (HeI = 21.2 eV) was used. In addition, for structural information, XRD measurements were recorded using a SIEMENS D5000 instrument. The thickness measurements was investigated using cross view of SEM (Hitachi S-4800 field emission at 5 kV).

## Electronic supplementary material

Supplementary material
