# Supplementary material for: Valence band behaviour of zirconium oxide, Photoelectron and Auger spectroscopy study
Source: Sci Rep. 2018 Nov 2;8:16251. doi: 10.1038/s41598-018-34570-w (PMC6214953; doi:10.1038/s41598-018-34570-w)
Supplement: Supplementary file 1 — Supplementary material [file 41598_2018_34570_MOESM1_ESM.pdf]

Supplementary Information

Valence band behaviour of zirconium oxide, Photoelectron and Auger spectroscopy study

Zakaria Azdad[1], Laurent Marot[1,*], Lucas Moser[1], Roland Steiner[1] and Ernst Meyer[1]

[1] Department of Physics, University of Basel, Klingelbergstrasse 82, 4056 Basel, Switzerland

* Corresponding author: laurent.marot@unibas.ch

Table of contents

1) Atomic Force Microscopy, X-ray diffraction and X-ray photoelectron spectroscopy of RF magnetron sputtering deposited films 1

2) Charge correction of XPS spectra 4

1) Atomic Force Microscopy, X-ray diffraction and X-ray photoelectron spectroscopy of RF magnetron sputtering deposited films

Figure S1 displays the change in the surface morphology of different thin films under different deposition conditions (oxygen percentage in the deposition chamber) measured using a Bruker Dimension 3100 atomic force microscope in tapping mode. The change in surface roughness is shown in the Figure S1 as an insert and is given by the root mean square described as: $\sqrt{\frac{1}{N}\sum_{i=1}^{N} y_i^2}$, where y is the interface height (nm) at a given point, taken N number of intervals across the interface.

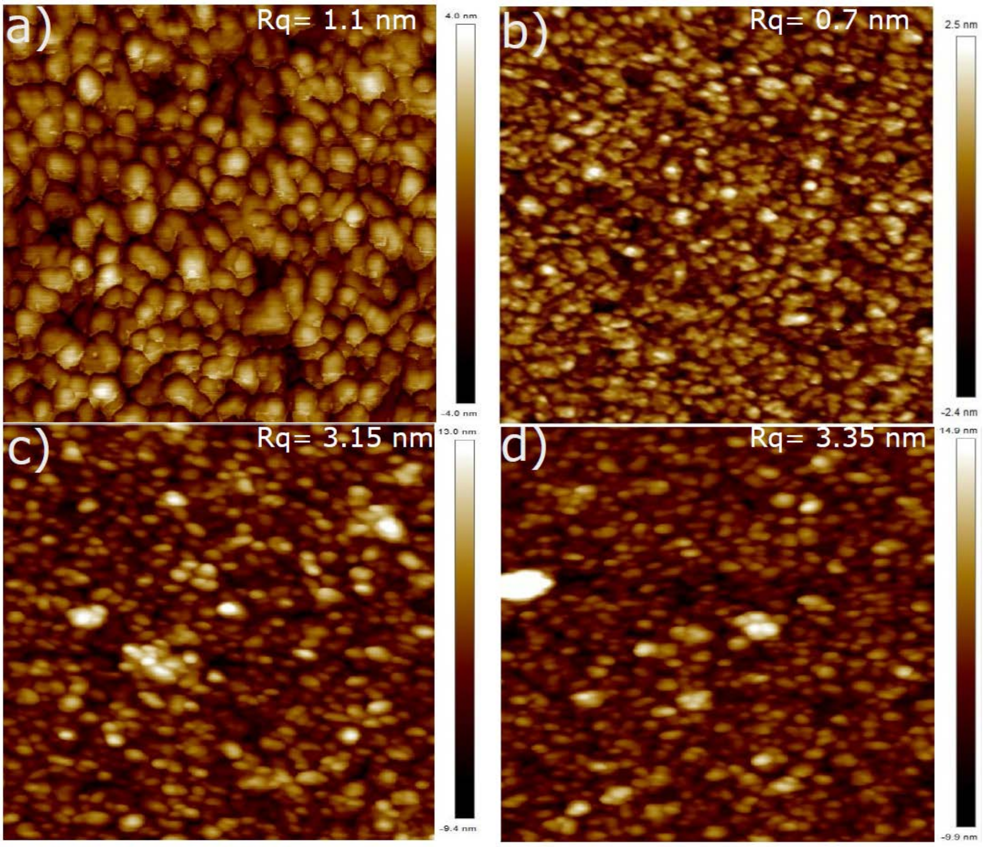

Figure S1: Evolution of the morphology of RF magnetron sputtering deposited films with $\frac{O}{Zr}$ = 1.3 for a) 4.5, b) 6, c) 9 and d) 11% of oxygen in the chamber. The image size is 500 × 500 nm.

X-rays diffractograms (XRD) were recorded using a SIEMENS D5000 instrument with monochromatic Cu Kα radiation (40 kV and 30 mA) at a glancing incidence of 5°. All XRD patterns were obtained with scan steps of 0.05° and with 8 seconds acquisition time in the range of 20 -90 °.

Figure S2 shows the XRD pattern of the deposited film. The thickness of the all the films was checked by Scanning Electron Microscope (SEM) cross section and evaluated to 35 ± 5 nm. All the films deposited shows a dominant monoclinic crystalline phase oriented along (-111) direction. The crystalline size was evaluated to 5 nm using Scherrer 's formula.

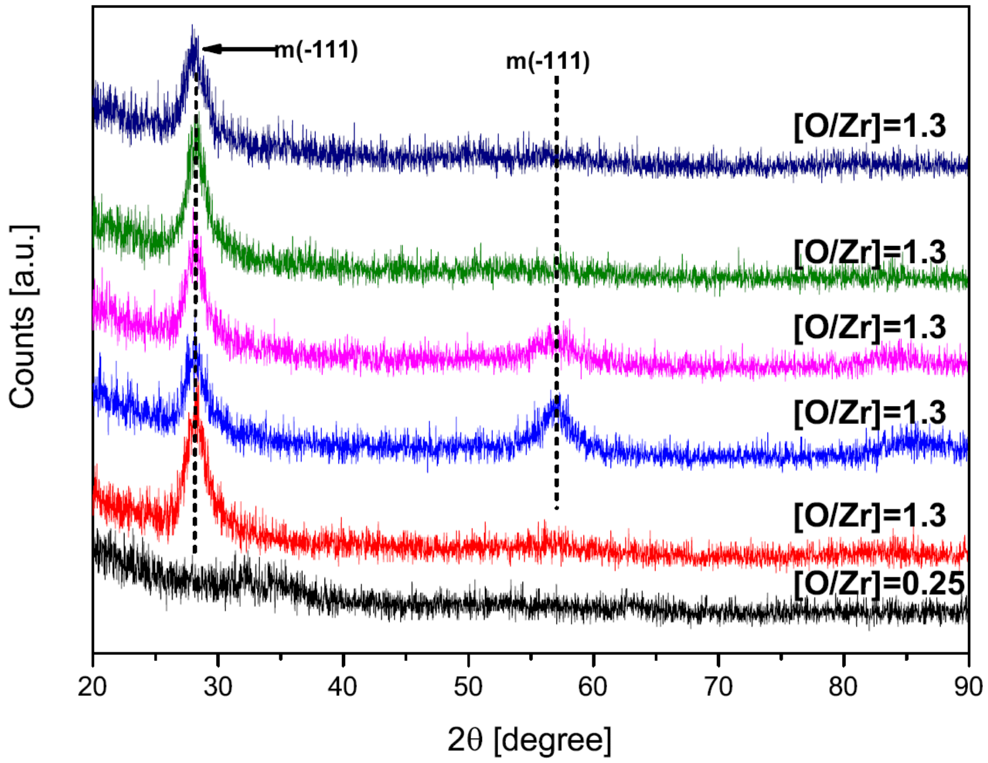

Figure S2: XRD patterns of deposited films using RF magnetron sputtering.

Figure S3 presents the X-ray Photolectron Spectroscopy (XPS) measurements of the same films before and after charge correction using MNV reference. All our samples were verified to be stable in ultra-high vacuum (UHV) and under X-ray irradiation. All the samples were measured with the same conditions (acquisition mode, step size and irradiation time).

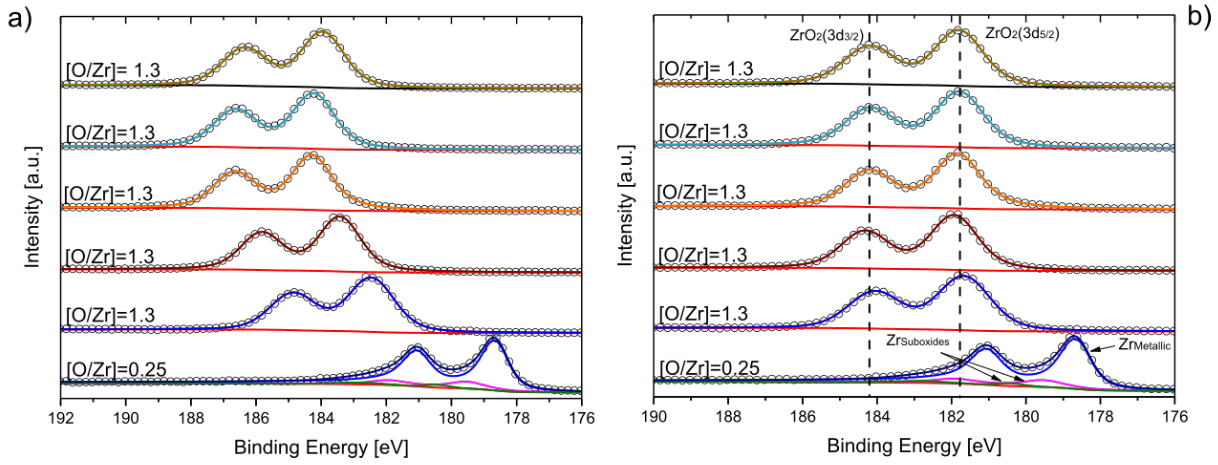

Figure S3: XPS of Zr3d core level spectra a) as measured after deposition and b) corrected using MNV reference. The colored lines represent the different components. The open circles stand for the spectrum data points after background subtraction. The solid lines behind the open circles are the fit sum of the components.

2) Charge correction of XPS spectra

Figure S4 displays the XPS core-level spectra of C1s, O1s and Zr3d of exposed films to air. The spectra were recorded on ESCALAB 210 spectrometer using monochromatic Al Kα radiation ($h\nu$ = 1486.6 eV). The Gaussian broadening of the spectrometer (convolution of the spectral resolution, which itself is a convolution of the analyzer and source resolution and thermal broadening was measured as 0.55 eV for the 20 eV analyzer pass energy. Normal electron escape angle and a step size of (maximum) 0.05 eV were used. Initially, wide scan XPS spectra from 0 to 1200 eV were taken in order to specify all the species present on the samples surface. The base pressure in the chamber was around 1×10^{-7} Pa during acquisition. The binding energy scale was calibrated using a clean gold sample and positioning the $Au4f_{7/2}$ line at 84.0 eV binding energy. As all the samples were verified to be stable in UHV and under X-ray irradiation, longer integration times could be used to optimize the signal to noise ratio.

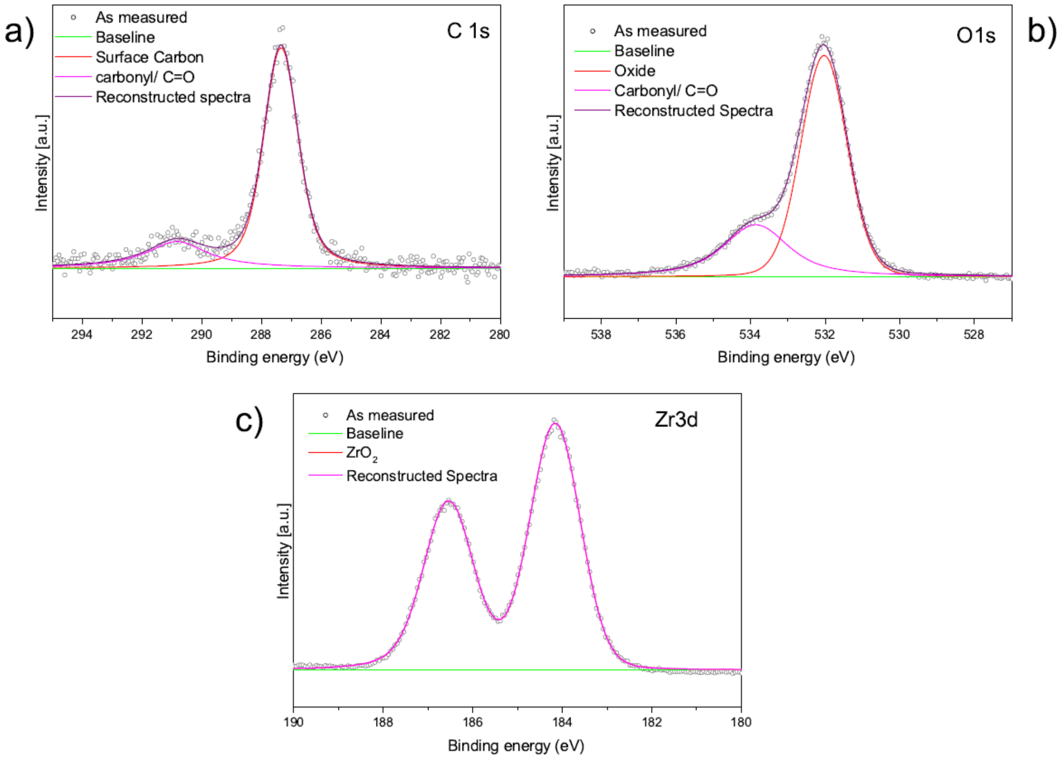

Figure S4: XPS core level spectra of a) C1s, b) O1s and c) Zr3d of film exposed to air. The colored lines represent the different components. The open circles stand for the spectrum data points after background subtraction. The solid lines behind the open circles are the fit sum of the components.

The as measured core-level spectra after gold deposition on ZrO_x film are shown in Figure S5. The XPS measurement confirms the absence of carbon trace in the freshly deposited film. The gold thin film was deposited on zirconia film without breaking the vacuum. Using a quartz micro-balance, the deposited gold thin film was estimated to 1 nm.

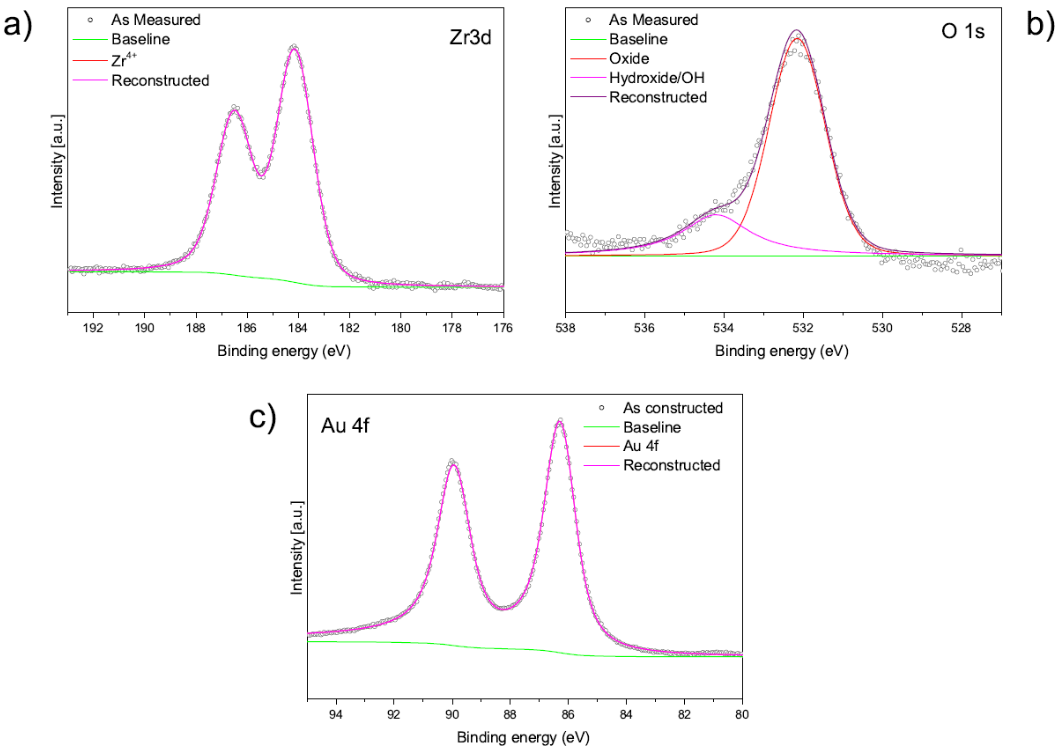

Figure S5: XPS core level spectra of a) Zr3d, b) O1s and c) O1s of the gold film deposited in top of zirconium. The colored lines represent the different components. The open circles stand for the spectrum data points after background subtraction. The solid lines behind the open circles are the fit sum of the components.

In Figure S6, SEM cross-sections of thick films are presented. The ratio $\frac{O}{Zr}$ is displayed in the insert.

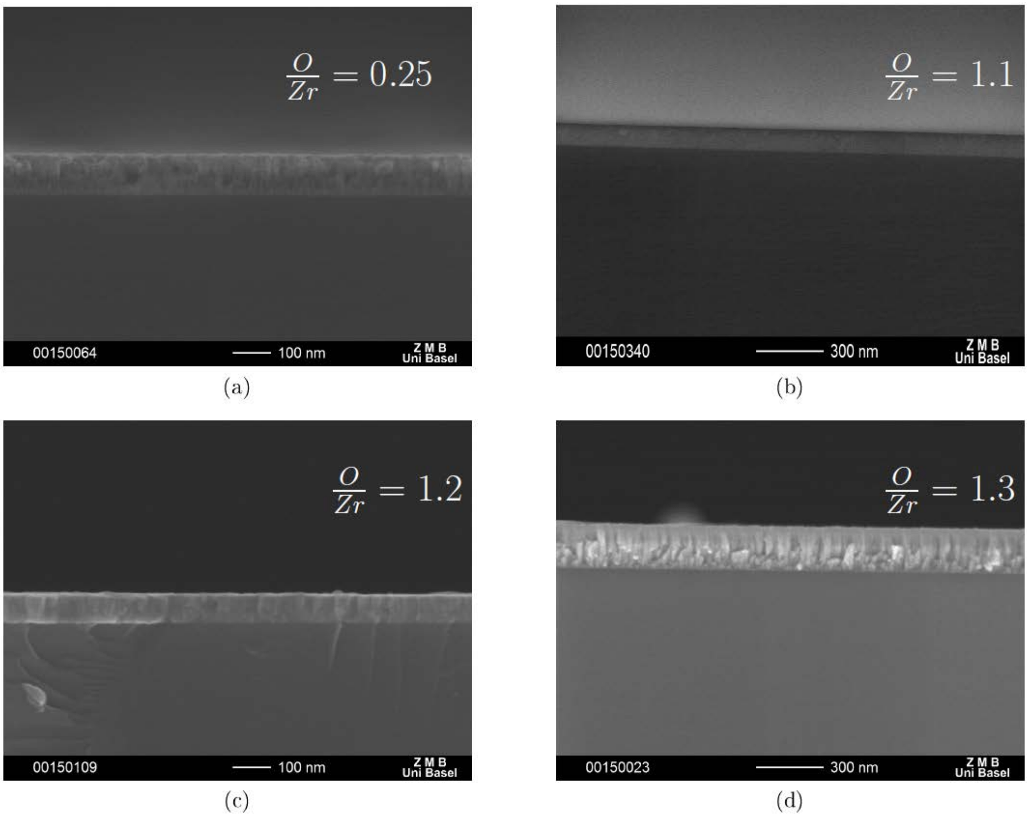

Figure S6: SEM cross-sections images.
